# Translational Informatics for Neuropharmacology: Databases, Ontologies, and Analytics

**DOI:** 10.2174/011570159X411123250509100901

**Published:** 2025-05-14

**Authors:** Bairong Shen, Nigel H. Greig, Mohammad Amjad Kamal

**Affiliations:** 1 Institutes for Systems Genetics, Frontiers Science Center for Disease-Related Molecular Network, West China Hospital, Sichuan University, Chengdu, 610041, China;; 2 Translational Gerontology Branch, National Institute on Aging, National Institutes of Health, Baltimore, MD 21224, USA;; 3 Department of Pharmaceutical Sciences, College of Pharmacy, Princess Nourah bint Abdulrahman University, Saudi Arabia; Department of Pharmacy, Daffodil International University, Bangladesh; Novel Global Community Educational Foundation, Australia

Advances in neuropharmacology, as a branch of pharmacology, have significantly increased over the past century, offering valuable insights into how the nervous system develops, is optimally maintained during healthy aging, and goes awry in neurological disorders. While only four useful neurological drugs were considered to exist prior to 1900 (morphine and aspirin for pain mitigation and management, nitrous oxide anesthesia in surgery, and caffeine for drowsiness), and four more drug classes were introduced by the 1950s (pethidine and analogs for pain, barbiturates and phenytoin for epilepsy, and antihistamines), hundreds of effective drugs from multiple drug classes are now approved by regulatory authority and widely used to mitigate neurological disorders worldwide. Although rewarding that such scientific progress has been made, the effective treatment of neurodegenerative and neuropsychiatric disorders, pain, and addictions remains severely wanting. A potential way to advance the neuropharmacology field is to apply current technological advances available from separate but parallel fields, particularly in relation to recent advances in translational informatics that have emerged as a pivotal field and hold the potential to bridge the treacherous gap between basic research and clinical application in neuropharmacology. A key focus of this thematic issue is to explore the advances, challenges, and applications of database management, ontologies, and analytics in the realm of neuropharmacology. In this special issue of *Current Neuropharmacology*, contributions from authors worldwide in the form of scientific manuscripts across the disciplines of neuropharmacology and informatics that are directed towards a greater understanding and treatment of neurological disorders are highly welcomed. In this regard, submissions are encouraged from basic and clinical researchers, practitioners, and experts/collaborative teams at the intersection of neuropharmacology and informatics. Our rationale is that accepted articles will hopefully increase our understanding of neurological disorders, improve drug discovery processes (both target identification and drug evaluation/development), and ultimately enhance patient outcomes through the application of translational informatics.

In an increasingly data-driven and artificial intelligence (AI)-supported era, translational informatics is a crucial discipline for translating basic medical and healthcare research into practical applications [1, 2]. In this regard, Bairong Shen and colleagues explored translational informatics-driven drug repositioning strategies for neurodegenerative diseases. Zheng *et al.* [1] highlighted computational models presently utilized in neurodegenerative disease research and identified databases that hold potential for future drug repositioning efforts. In the present AI period, drug repositioning, as a data-driven strategy, offers a promising avenue for developing treatments suited to the complex and multifaceted nature of neurodegenerative diseases. These advancements could furnish patients with more rapid, cost-effective therapeutic options [1].

To best harness available science and direct it to improve human health involves the use of databases for complex systems, ontologies for data standardization [3], and the creation of knowledge graphs for explainable AI [4]. Understanding the molecular mechanisms of diseases is fundamental for comprehending the personalized and heterogeneous nature of medical conditions [5, 6]. This special issue on translational informatics, therefore, covers a broad spectrum of research areas within neuropharmacology from the perspective of informatics and molecular mechanisms.

Mittal *et al.* investigated how ontologies can act as pioneers in precision medicine for neurodegenerative diseases, capitalizing on neural networks of knowledge [7]. Their review focused on how ontologies are revolutionizing precision medicine to understand neurodegenerative illnesses. Ontologies, which are structured frameworks that outline the relationships between concepts in a certain field, offer a crucial foundation for combining different biological data. Novel insights into the construction of a precision medicine approach to treat neurodegenerative diseases are aided by growing advancements in the area of pharmacogenomics. In this regard, it is interesting to examine how semantic data can be combined with cutting-edge computer methods, such as AI and machine learning, to make brain disease diagnostic and prediction models more exact and accurate [7].

Luis Tovar-y-Romo and collaborators evaluated how the expanding field of neuroproteomics can help identify potential biomarkers to mitigate disease development. In detail, Bernal-Vicente *et al.* [8] concentrated on neuroprotective proteins found in extracellular vesicles that originate from hypoxia-stressed astrocytes. Their findings highlighted the role of astrocyte-derived extracellular vesicles in promoting neuronal survival under hypoxic conditions. Extracellular vesicles may contribute to neuroprotection and support recovery following cerebral ischemia and provide a database for future investigations involving astrocyte-derived extracellular vesicles [8]. The discussion further explores how such databases can be leveraged to best develop potential biomarkers and/or drug targets for disease prevention.

Migraine is a prevalent and debilitating neurological disorder, and current therapies are frequently ineffective and have adverse effects. Recent studies on neuropharmacology have highlighted the serotonin 1B receptor (HTR1B) receptor as a viable target for migraine treatment since it influences pain and vasoconstriction [9]. In this light, Imran Zafar and his team employed an informatics-based methodology to target the HTR1B pathways as a migraine treatment strategy. Ahmad *et al.* [9] found that the toxicity risk assessment of xaliproden was favorably modest, which provides a foundation for targeted HTR1B-based migraine therapies and highlights the value of informatics tools in accelerating drug discovery in neuropharmacology [9].

Elliot Glotfelty and associates analyzed Nogo-family genes in omics datasets of mouse and human microglia, identifying LINGO1 as a potentially viable drug target in Alzheimer's disease (AD). Glotfelty *et al.* [10] identified differential expression of Nogo-family genes across age, sex, and disease/injury in mice. To analyze human microglia, they utilized a new tool, the CZ CellxGene Discover, to aggregate 21 single-cell sequencing datasets of human brain cells in AD patients and age-matched control subjects. In humans, Lingo1 is highly enriched in human AD microglia, a previously undescribed finding. Glotfelty *et al.* then used The Alzheimer’s Cell Atlas (TACA) to further verify whether this enrichment correlates to disease state, severity of human AD diagnosis, or sex of patients. This current work provides a comprehensive analysis of Nogo-family genes in microglia and identifies Lingo1 as an interesting potential novel therapeutic target for AD [10].

Regarding diagnosis, Mohammad Kamal and colleagues assessed the utilization of AI-enhanced MRI to advance the diagnosis of AD, systematically discussing the associated challenges and implications. In this regard, Batool *et al.* [11] provided a comprehensive summary of deep learning studies, critically evaluating their methodologies and outcomes. By categorizing the studies into various sub-fields, they highlighted the strengths and limitations of using MRI-based deep learning approaches for diagnosing brain disorders. Furthermore, they discussed the potential implications of these advancements in clinical practice, considering the challenges and future directions for improving diagnostic accuracy and patient outcomes. The findings of this detailed analysis contribute to the ongoing efforts in harnessing AI to achieve a better understanding and management of AD [11].

In the context of disease intervention and treatment, Bashir Ullah and his group examined both traditional and emerging drug-targeting sites, along with the pivotal role of translational informatics in AD. Specifically, Khan *et al.* [12] discussed the role of translational informatics involving data modeling, predictive analytics, explainable AI, and multimodal approaches in the management and prediction of AD. Their article can serve as a guide for future research efforts in the fields of neuroscience, neuropharmacology, drug delivery sciences, and translational informatics [12].

Muzamil Ahmad and co-researchers explored the latest progress in neuropharmacological interventions of plant origin for Parkinson's disease (PD) by evaluating the therapeutic potential of traditional herbal formulations alongside computational research methods. In this regard, Muzaffer *et al.* [13] examined the mechanisms of action, synergistic effects, and implications for traditional herbal formulations in relation to PD management. The significance of computational techniques, such as molecular docking, molecular dynamics simulations, pharmacophore modeling, and network pharmacology, were also evaluated. This review article additionally assessed how traditional wisdom aligns and integrates with modern scientific inquiry in relation to herbal-based interventions for PD [13].

Overall, this special issue underscores the importance of translational informatics, encompassing databases, ontologies, and AI-enabled analytics in relation to improving our understanding of neuropharmacology focused on improving our diagnosis and/or treatment of neurological disorders. It illustrates how these components can successfully bridge the gaps between understanding molecular disease mechanisms, enhancing diagnostic techniques, and formulating innovative therapeutic approaches in neuropharmacology. Collaborative efforts that combine computational and experimental methods are indispensable for accelerating the development of diagnostics and targeted therapies.

It has been a pleasure for the guest editors to work with both the Editor-in-Chief, Professor Ferdinando F. Nicoletti, and the senior publication manager, Miss Rabia Moin, of *Current Neuropharmacology*, and we are honored by the opportunity of publishing this thematic issue in such a prestigious international journal. In doing so, we would like to thank the authors of all the included articles for their thoughtful contributions to the scientific literature and to acknowledge the significant contributions of others who reviewed, edited, and processed the manuscripts to ensure their high quality at the time of publication.
